# Epsilon-aminocaproic acid versus tranexamic acid in total knee arthroplasty: a meta-analysis study

**DOI:** 10.1186/s10195-019-0534-2

**Published:** 2019-07-18

**Authors:** Osman Riaz, Adeel Aqil, Samir Asmar, Raees Vanker, James Hahnel, Christopher Brew, Richard Grogan, Graham Radcliffe

**Affiliations:** Bradford Teaching Hospitals NHS Foundation Trust, Duckworth Lane, Bradford, BD9 6RJ UK

**Keywords:** Tranexamic acid, Epsilon-aminocaproic acid, Total knee arthroplasty

## Abstract

**Introduction:**

Total knee arthroplasty (TKA) surgery can be associated with significant blood loss. Among the problems associated with such blood loss is the need for transfusions of banked blood [1]. Transfusions not only have a financial consequence but also carry a small risk of disease transmission to the patient. Antifibrinolytics have been successfully used to reduce transfusion requirements in elective arthroplasty patients. The objective of this meta-analysis is to determine which of tranexamic acid (TXA) and epsilon-aminocaproic acid (EACA) is more effective for reducing peri-operative blood loss, and lessening the need for blood transfusion following knee arthroplasty surgery.

**Materials and methods:**

MEDLINE, Embase and CINAHL databases were searched for relevant articles published between January 1980 to January 2018 for the purpose of identifying studies comparing TXA and EACA for TKA surgery. A double-extraction technique was used, and included studies were assessed regarding their methodological quality prior to analysis. Outcomes analysed included blood loss, pre- and post-operative haemoglobin, number of patients requiring transfusion, number of units transfused, operative and tourniquet time, and complications associated with antifibrinolytics.

**Results:**

Three studies contributed to the quantitative analysis of 1691 patients, with 743 patients included in the TXA group and 948 in the EACA group. Estimated blood loss was similar between the two groups [95% confidence interval (CI) −0.50, 0.04; *Z* = 1.69; *P* = 0.09]. There were no differences between the two groups regarding the percentage of patients requiring transfusion (95% CI 0.14, 4.13; *Z* = 0.31; *P* = 0.76). There was no difference in the pre- and post-operative haemoglobin difference between the two groups (95% CI −0.36, 0.24; *Z* = 0.38; *P* = 0.70). There was no difference in the average number of transfused units (95% CI −0.53, 0.25; *Z* = 0.71; *P* = 0.48). There was no difference in the operative (95% CI −0.35, 0.36; *Z* = 0.04; *P* = 0.97) or tourniquet time (95% CI −0.16, 0.34; *Z* = 0.72; *P* = 0.47). Similarly, there was no difference in the percentage of venous thromboembolism between the two groups (95% CI 0.17, 2.80; *Z* = 0.51; *P* = 0.61).

**Conclusions:**

This study did not demonstrate TXA to be superior to EACA. In fact, both antifibrinolytic therapies demonstrated similar efficacy in terms of intra-operative blood loss, transfusion requirements and complication rates. Currently EACA has a lower cost, which makes it an appealing alternative to TXA for TKA surgery.

**Level of evidence:**

3.

## Introduction

Total knee arthroplasty (TKA) surgery can be associated with significant blood loss, which can be associated with a number of problems, including the requirement for blood transfusions [1]. Blood transfusions incur significant cost in terms of materials and technician and nursing staff and pose significant risk of adverse clinical outcomes. These risks include transfusion-related infections, intravascular haemolysis, transfusion-induced coagulopathy, renal impairment, immunologic incompatibility and even mortality [2]. In addition to its association with blood transfusion, excessive bleeding can impair the result of TKA surgery through haematoma, swelling, stiffness, prolonged use of drains, increased length of stay, delayed functional recovery and rehabilitation [3, 4].

Antifibrinolytics have already been successfully used to reduce transfusion requirements in elective arthroplasty patients. Tranexamic acid (TXA) and epsilon-aminocaproic acid (EACA) are two agents that have been shown to reduce peri-operative surgical blood loss and transfusion requirements [5–10]. EACA is a lysine analog class of antifibrinolytic which has been widely used in cardiac procedures and has only recently been used in elective orthopaedic surgery [11, 12]. Whilst studies have demonstrated EACA to have a good efficacy and safety profile [13], TXA has been preferentially used in orthopaedics. This may in part be due to the familiarity of TXA to orthopaedic surgeons and anaesthetists and the fact that it was the first to be introduced onto the market. However, TXA appears to be considerably more expensive than its newer counterpart [14, 15].

The objective of this meta-analysis is to determine which of TXA or EACA has greater efficacy for reducing peri-operative blood loss and transfusion requirements associated with knee arthroplasty surgery. The secondary objective is to compare complication rates between the two drugs.

## Materials and methods

**Fig. 1:**
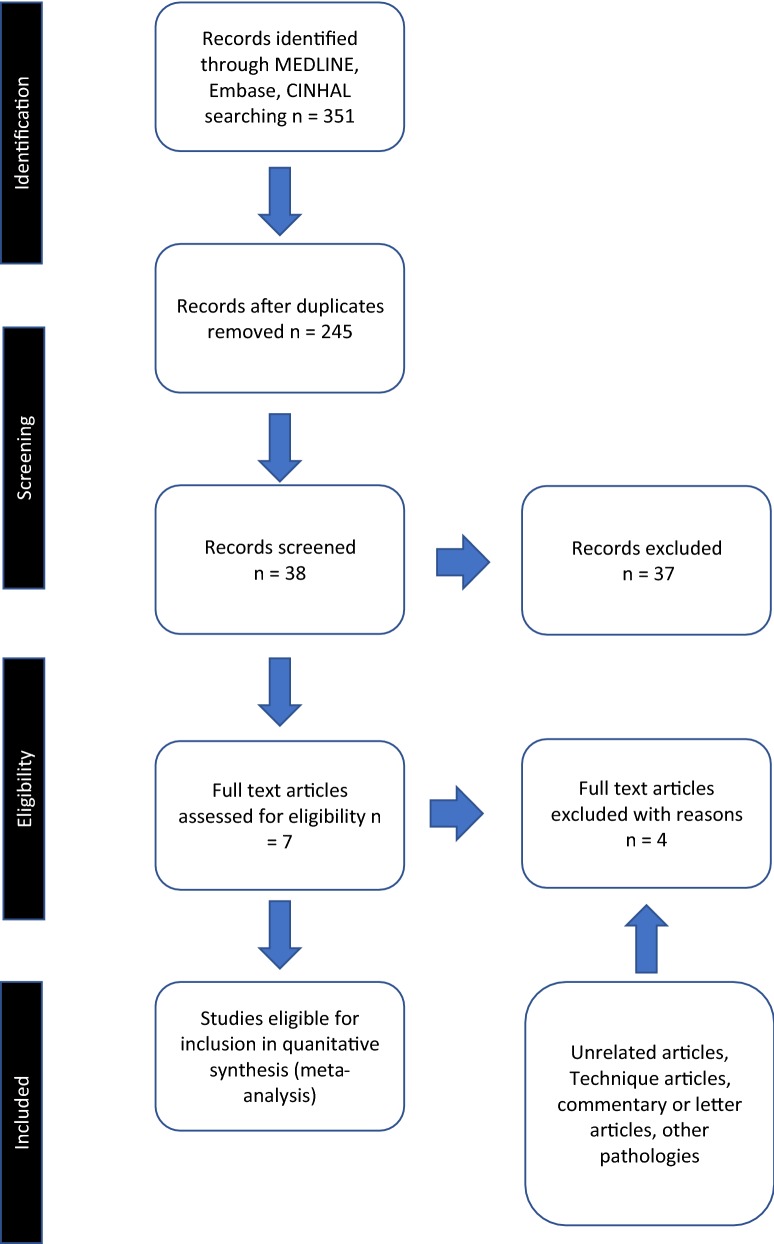
PRISMA flowchart of study selection

A comprehensive search of the published literature in MEDLINE, Embase and CINAHL databases was performed for articles comparing EACA and TXA during total knee arthroplasty. The text words “aminocaproic acid”, “EACA”, “tranexamic acid”, and “TXA”, were used in combination with the medical subject headings “knee arthroplasty” and “knee replacement”. All included studies directly compared EACA and TXA for total knee arthroplasty. In addition, patients were of minimum age of 18 years, and comprised both sexes. Irrelevant articles and studies, which failed to meet inclusion criteria, such as reviews, evident from their titles and abstract, were excluded. Nonclinical studies, or those assessing other forms of arthroplasty such as hip, were excluded. Similarly, studies not directly comparing EACA with TXA were also excluded. Relevant articles referenced in these publications were obtained, and the “related article” function was used to widen the results. No language restriction was applied. All abstracts, comparative studies, and citations were searched comprehensively. This study conformed to QUOROM and PRISMA guidelines [16, 17]. A flowchart of the literature search is provided in Fig. 1. The quality of included trials was rated using the Scottish Intercollegiate Guidelines Network scoring system (Table 1) [18].

**Table 1:** Methodological quality of prospective studies included, adapted from the Scottish Intercollegiate Guidelines Network

Quality variable	Boese et al. (2017) [19]	Camarasa et al. (2006) [20]	Churchill et al. (2017) [21]
1.1 The study addresses an appropriate and clearly focussed question (Y/N/can’t say)	Y	Y	Y
1.2 The two groups being studied are selected from source populations that are comparable in all respects other than the factor under investigation (Y/N/can’t say)	Y	Y	Y
1.3 The study indicates how many of the people asked to take part did so, in each of the groups being studied (Y/N/NA)	Y	Y	Y
1.4 The likelihood that some eligible subjects might have the outcome at the time of enrolment is assessed and taken into account in the analysis (Y/N/NA)	N	N	N
1.5 What percentage of individuals or clusters recruited into each arm of the study dropped out before the study was completed	10%	10%	0%
1.6 Comparison is made between full participants and those lost to follow-up, by exposure status (Y/N/can’t say/NA)	N	N	NA
1.7 The outcomes are clearly defined (Y/N/can’t say)	Y	Y	Y
1.8 The assessment of outcome is made blind to exposure status. If the study is retrospective this may not be applicable (Y/N/can’t say/NA)	Y	Y	N
1.9 Where blinding was not possible, there is some recognition that knowledge of exposure status could have influenced the assessment of outcome (Y/N/can’t say)	N	N	N
1.10 The method of assessment of exposure is reliable (Y/N/can’t say)	Y	Y	Y
1.11 Evidence from other sources is used to demonstrate that the method of outcome assessment is valid and reliable (Y/N/can’t say/NA)	Y	Y	Y
1.12 Exposure level or prognostic factor is assessed more than once (Y/N/can’t say/NA)	N	N	N
1.13 The main potential confounders are identified and taken into account in the design and analysis	Y	Y	Y
1.14 Have confidence intervals been provided where appropriate?	Y	Y	Y
How well was the study done to minimise the risk of bias or confounding? (high quality = ++, acceptable = +, unacceptable = −)	+++	+++	++
Taking into account clinical considerations, your evaluation of the methodology used, and the statistical power of the study, do you think there is clear evidence of an association between exposure and outcome? (Y/N/can’t say)	Y	Y	Y
Are the results of this study directly applicable to the patient group targeted in this guideline? (Y/N)	Y	Y	Y

We found three articles which met our inclusion criteria [19–21]. Each article was critically reviewed by two researchers (O.R., A.A.) using a double-extraction method for eligibility. Article extraction was performed independently, and any conflict resolved before final analysis.

Outcome measures were chosen if they were comparable to those from other papers. The primary outcome measures included blood loss and the amount of tranfusion required. Secondary objectives compared operative time, tourniquet time and complications between the two drug groups.

Statistical analyses were performed using Review Manager version 5.0 (The Nordic Cochrane Centre Copenhagen, Denmark). *P*-value less than 0.05 was chosen as the significance level for outcome measures. For continuous data, the inverse variance method was used for the combination of standardised mean differences (SMDs). Binary data were summarised as risk ratios (RR) and combined using the Mantel–Haenszel method. In each case, a heterogeneity test was performed and a random-effects model used for analyses for consistency and to deal with possible population heterogeneity. Forest plots were used for graphical representation. Funnel plots were not constructed, as they were deemed inappropriate for this number of studies.

## Results

Three studies met the inclusion criteria. All three studies reported outcomes which were suitable for quantitative analysis. In total, data from 1691 patients were used in the analysis. There were 743 patients in the TXA group and 948 patients in the EACA group.

### Estimated blood loss

**Fig. 2:**
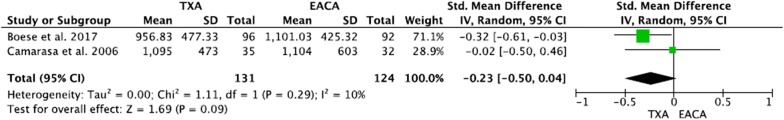
Estimated total blood loss

Two studies contributed to the analysis of estimated blood loss [19, 20]. There was no significant heterogeneity between the two trials (*df* = 1; *I*^2^ = 10%; *Q* = 1.11; *P* = 0.29). There was no significant difference in estimated blood loss between TXA and EACA in these two studies (95% CI −0.50, 0.04; *Z* = 1.69; *P* = 0.09) (Fig. 2).

### Percentage of patients requiring transfusion

**Fig. 3:**
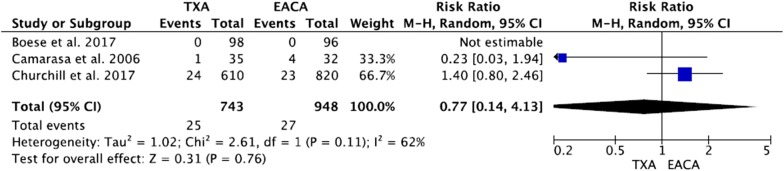
Percentage of patients requiring transfusion

Three studies contributed to the analysis of the percentage of patients requiring transfusion [19–21]. There was no significant heterogeneity between the three trials (*df* = 1; *I*^2^ = 62%; *Q* = 2.61; *P* = 0.11). There was no significant difference in the percentage of patients requiring transfusion between TXA and EACA groups in these three studies (95% CI 0.14, 4.13; *Z* = 0.31; *P* = 0.76) (Fig. 3).

### Difference between pre- and post-operative haemoglobin (Hb)

**Fig. 4:**
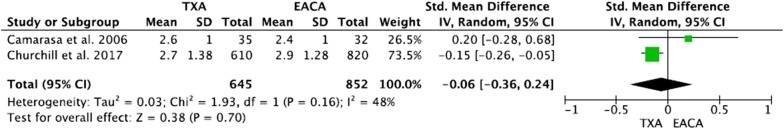
Difference in pre- and post-operative Hb

Two studies contributed to the analysis of difference between pre- and post-operative haemoglobin levels [20, 21]. There was no significant heterogeneity between these studies (*df* = 1; *I*^2^ = 48%; *Q* = 1.93; *P* = 0.16). There was no significant difference in the pre- and post-operative haemoglobin change between TXA and EACA groups (95% CI −0.36, 0.24; *Z* = 0.38; *P* = 0.70) (Fig. 4).

### Average number of transfused units

**Fig. 5:**
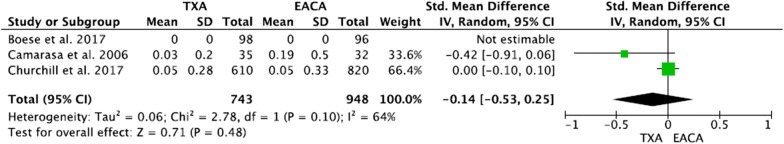
Average no. of transfused units

Whilst three studies reported on this outcome, only data from two studies were comparable in the analysis [20, 21]. There was some heterogeneity between these trials, justifying the use of a random-effects model (*df* = 1; *I*^2^ = 64%; *Q* = 2.78; *P* = 0.10). There was no significant difference in the average number of transfused units between TXA and EACA groups (95% CI −0.53, 0.25; *Z* = 0.71; *P* = 0.48) (Fig. 5).

### Operative time in minutes

**Fig. 6:**
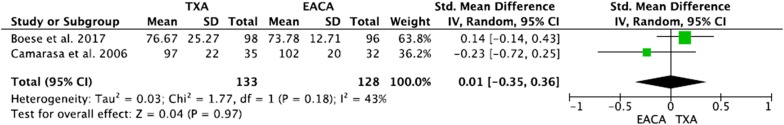
Operative time in minutes

Two studies contributed to the analysis of operative time [19, 20]. There was some heterogeneity between the two trials, justifying the use of a random-effects model (*df* = 1; *I*^2^ = 43%; *Q* = 1.77; *P* = 0.18). There was no significant difference in operative time between TXA and EACA groups (95% CI −0.35, 0.36; *Z* = 0.04; *P* = 0.97) (Fig. 6).

### Tourniquet time in minutes

**Fig. 7:**
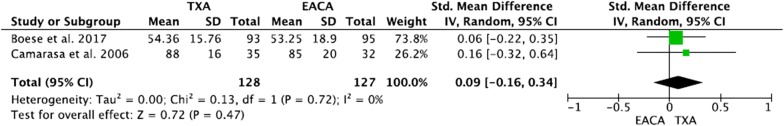
Tourniquet time in minutes

Two studies contributed to the analysis of tourniquet time [19, 20]. There was no significant heterogeneity between the two trials (*df* = 1; *I*^2^ = 0%; *Q* = 0.13; *P* = 0.72). There was no significant difference in tourniquet time between TXA and EACA groups (95% CI −0.16, 0.34; *Z* = 0.72; *P* = 0.47) (Fig. 7).

### Percentage of patients with VTE complications

**Fig. 8:**
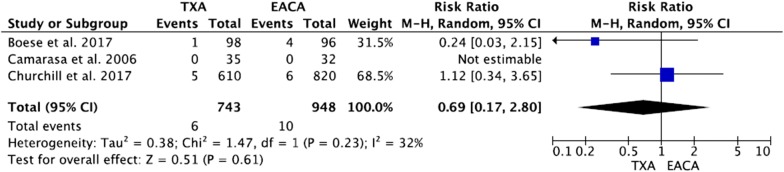
Percentage of patients with VTE events

Three studies contributed to the analysis of patients with VTE events [19–21]. There was no significant heterogeneity between the three trials (*df* = 1; *I*^2^ = 32%; *Q* = 1.47; *P* = 0.23). There was no significant difference in VTE events between TXA and EACA groups in these three studies (95% CI 0.17, 2.80; *Z* = 0.51; *P* = 0.61) (Fig. 8).

## Discussion

Excessive intraoperative blood loss can predispose to hypotension and other cardiac, pulmonary and renal complications, with a subsequent knock-on adverse effect on peri-operative recovery. Excessive bleeding in the early post-operative period can be associated with haematoma formation, swelling, stiffness and wound complications, which may impair the long-term outcome of TKA surgery. In addition, post-TKA blood transfusions have been reported to cause acute lung injury, infections, immune suppression and hypersensitivity reactions [22].

The normal process of fibrinolysis involves the liquefaction of fibrin within blood clots. It consists of two steps: degradation of fibrin and activation of plasminogen [23]. Antifibrinolytic agents such as TXA and EACA decrease blood loss by attenuating this fibrinolytic process [6–8].

Specifically, TXA works by competitively binding to plasminogen lysine receptor sites, preventing formation of plasmin and its action in degrading fibrin. At high doses, it can also have a protective effect on platelets. EACA is a 6-aminohexanoic acid also belonging to the lysine class of antifibrinolytic agents. Thus, EACA is believed to work by a similar mechanism to TXA.

TXA has gained in popularity and is in routine use in many centres due to its efficacy in reducing transfusion risks and costs [24]. Many studies have demonstrated the benefits of TXA in reducing peri-operative blood loss and transfusion requirements. However, fewer studies have investigated EACA in elective orthopaedic surgery, and even fewer compare the two drugs head to head in large enough numbers to draw any meaningful conclusions [25–27]. Given that these drugs work in a similar way and have the same indications, it seems reasonable to perform a quantitative analysis of all studies comparing these two drugs, in order to gain statistical power and make more meaningful conclusions. To the best of the authors’ knowledge, this is the first meta-analysis comparing the outcomes and complications of TXA and EACA in elective TKA surgery.

In this meta-analysis of 1691 total knee arthroplasty patients, we found no statistical difference between TXA and EACA with regards to blood loss, transfusion requirements or VTE complication rates. These findings are in accordance with other recent reviews of these drugs in patients undergoing other orthopaedic procedures [28–30].

Clearly, EACA may prove to be a useful alternative in case of patient allergies to TXA. However, one study suggested that it should be considered as first-line therapy, given that EACA is more cost effective that its counterpart TXA. Whilst the singular difference in cost was only £26, the cumulative cost saving to a healthcare provider could be quite significant [22]. As it appears that the efficacy and complication rates of the two drugs are similar, it would seem reasonable for users to consider EACA favourably whilst it remains less expensive.

We acknowledge some limitations to this study. The studies included in the meta-analysis were of variable quality. However, following quantitative assessment, they were found to meet an acceptable standard prior to their inclusion. We also acknowledge the possibility of publication bias. This is difficult to account for with the number of studies included in this analysis, and readers should bear this in mind. In addition, there will have been some variability in transfusion protocols and population heterogeneity between studies. Thus, as a standard, we performed heterogeneity calculations to give clarity to the reader and used a random-effects model as standard to reduce any heterogeneity effects.

Whilst in individual studies, TXA and EACA were given at set times and set doses, these were different between studies. Whilst this creates some heterogeneity between studies, the slight difference between the dosage regimes will have little impact on the outcome variables, as all dosage regimes had similar outcomes across the studies.

This study is also limited by the fact we did not factor in cost-effectiveness analysis of the drugs used. This was because such data were not available from the published studies, and drug costs are likely to vary from region to region based on the healthcare system and whether cost reduction from bulk ordering can be achieved. We recommend that healthcare institutions investigate drug costs available to them when deciding which of these drugs to use.

Given the results of this study of 1691 patients, it can, however, be concluded that use of TXA and EACA was equally safe and effective as prophylaxis for excessive bleeding in TKA surgery. As EACA has lower cost, it may be seen as a suitable alternative first-line agent in order to reduce the financial burden on healthcare providers.

## Data Availability

Not applicable.

## Abbreviations

TKA: total knee arthroplasty
TXA: tranexamic acid
EACA: epsilon-aminocaproic acid
SMDs: standardised mean differences
Hb: haemoglobin
